# Identification of Transcription Factors of Santalene Synthase Gene Promoters and *SaSSY* Cis-Elements through Yeast One-Hybrid Screening in *Santalum album* L.

**DOI:** 10.3390/plants13131882

**Published:** 2024-07-08

**Authors:** Yunqing Zhou, Xiang Li, Dongli Wang, Zequn Yu, Yunshan Liu, Lipan Hu, Zhan Bian

**Affiliations:** 1 College of Biology and Food Engineering, Chongqing Three Gorges University, Chongqing 404100, China; zhouyunqing824@163.com (Y.Z.); lx541307@163.com (X.L.); 2 Guangdong Provincial Key Laboratory of Applied Botany, South China Botanical Garden, Chinese Academy of Sciences, Guangzhou 510650, China; 3 Key Laboratory of National Forestry and Grassland Administration on Plant Conservation and Utilization in Southern China, South China Botanical Garden, Chinese Academy of Sciences, Guangzhou 510650, China; 4 Key Laboratory of South China Agricultural Plant Molecular Analysis and Genetic Improvement, South China Botanical Garden, Chinese Academy of Sciences, Guangzhou 510650, China; 5 State Key Laboratory of Tree Genetics and Breeding, Research Institute of Tropical Forestry, Chinese Academy of Forestry, Guangzhou 510520, China; wangdongli1997@163.com (D.W.); lys061949@163.com (Y.L.); 6 Shanghai Gardening-Landscaping Construction Co., Ltd., Shanghai 200333, China; zequn2020@126.com

**Keywords:** cDNA library, promoter, sandalwood, *SaSSY* gene

## Abstract

The main components of sandalwood heartwood essential oil are terpenoids, approximately 80% of which are α-santalol and β-santalol. In the synthesis of the main secondary metabolites of sandalwood heartwood, the key gene, santalene synthase (*SaSSY*), can produce α-santalene and β-santalene by catalyzed (E, E)-FPP. Furthermore, santalene is catalyzed by the cytochrome monooxygenase SaCYP736A167 to form sandalwood essential oil, which then produces a fragrance. However, the upstream regulatory mechanism of the key gene santalene synthase remains unclear. In this study, *SaSSY* (*Sal3G10690*) promoter transcription factors and *SaSSY* cis-elements were screened. The results showed that the titer of the sandalwood cDNA library was 1.75 × 10^7^ CFU/mL, 80% of the inserted fragments identified by PCR were over 750 bp in length, and the positivity rate of the library was greater than 90%. The promoter region of the *SaSSY* gene was shown to have the structural basis for potential regulatory factor binding. After sequencing and bioinformatics analysis, we successfully obtained 51 positive clones and identified four potential *SaSSY* transcriptional regulators. Sal6G03620 was annotated as the transcription factor MYB36-like, and Sal8G07920 was annotated as the small heat shock protein HSP20 in sandalwood. Sal1G00910 was annotated as a hypothetical protein of sandalwood. Sal4G10880 was annotated as a homeobox-leucine zipper protein (ATHB-15) in sandalwood. In this study, a cDNA library of sandalwood was successfully constructed using a yeast one-hybrid technique, and the transcription factors that might interact with *SaSSY* gene promoters were screened. This study provides a foundation for exploring the molecular regulatory mechanism involved in the formation of sandalwood heartwood.

## 1. Introduction

Sandalwood (*Santalum album* L.) is a tropical tree with slow growth and semiparasitic characteristics. Sandalwood heartwood and sandalwood essential oil extracted from sandalwood have high economic and medicinal value. The aromatic heartwood of sandalwood has various applications in perfumes, religions, cosmetics, wood carving, and pharmacology [1,2]. The main aromatic substances in sandalwood essential oil are α-santalol and β-santalol, which are sesquiterpenoids and are mainly synthesized by the mevalonic acid (MVA) or non-mevalonic acid pathway. In addition, sandalwood essential oil also contains small amounts of α-sandalene, β-sandalene, trans-α-bergamot, and other components [3]. *SaSSY* is a key enzyme in the MVA synthesis pathway that produces α-santalene and β-santalene by catalyzed (E, E)-FPP. It is further catalyzed by the cytochrome monooxygenase SaCYP736A167 to produce santalol [4]. Santalol content is influenced by santalene synthases in sandalwood heartwood [5]. Terpenoids are the main substances of sandalwood heartwood essential oil. Terpenoids not only are the largest family of plant secondary metabolites but also play an important role in plant life [6]. Terpenoids are involved in plant defense against biological and abiotic stresses [7,8], attraction to pollinators [9], and plant adaptation to complex environments in plants [8,10]. *SaSSY* is an orthologous terpene synthase (*TPS*) gene, which is responsible for the production of key fragrant compounds in sandalwood [4]. Most TFs related to plant secondary metabolites can initiate the expression of specific genes encoding enzymes involved in biochemical pathways [6]. Transcription factors such as MYB, WRKY, bHLH, AP2/ERF, and bZIP have important regulatory effects on plant terpenoids [11]. The interaction between *SlMYB75* and *SlTPS12*, *SlTPS31*, and *SlTPS35* genes can moderately increase the accumulation of sesquiterpenes in *Solanum lycopersicum* [12]. The binding of Arabidopsis *MYC2* to the sesquiterpene synthase genes *pTPS21* and *pTPS11* will increase emission of sesquiterpenes, especially (E)-β-caryophyllene [13]. In peaches (*Prunus persica*), *PpERF61* activates the transcription of *PpTPS1* and *PpTPS3*, which increases the content of linalool [14]. *PpbHLH1* can activate the expression of *PpTPS3* in peaches, resulting in a significant increase in flavor related linalool production [15]. Nevertheless, until now, the *SaSSY* gene promoter and the TF acting on the *SaSSY* gene promoter have remained unclear.

This study aims to analyze the *SaSSY* promoter and identify transcription factors that specifically bind to the *SaSSY* promoter using yeast one hybrid system. The results of this study lay a foundation for further analysis of the molecular regulatory mechanism of sandalwood heartwood formation.

## 2. Results

### 2.1. RNA Extraction and Homogenization of Double-Stranded cDNA

**Figure 1:**
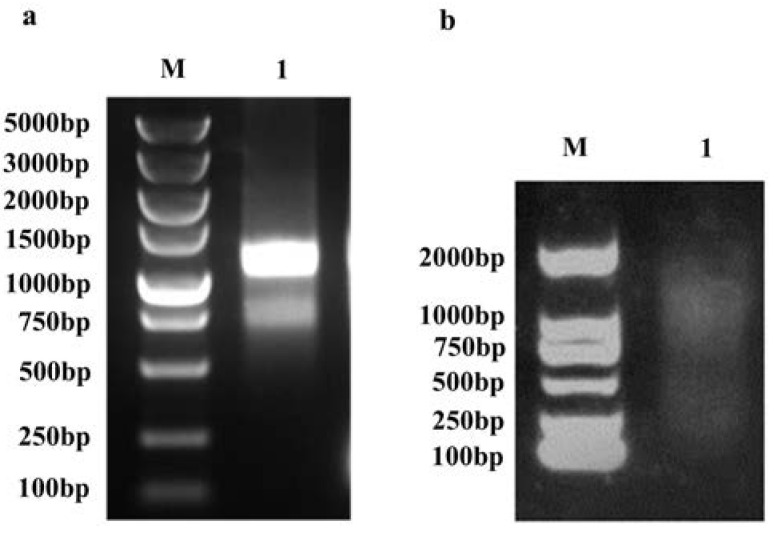
RNA agarose gel electrophoresis. (**a**) Agarose gel electrophoresis of RNA extracted from tissues; marker (5000/3000/2000/1500/1000/750/500/250/100 bp); Lane 1: total RNA. (**b**) Agarose gel electrophoresis of purified mRNA; marker (2000/1000/750/500/250/100 bp); Lane 1: mRNA.

The root, stem, and leaf of the sandalwood seedlings were collected for total RNA extraction. The results showed an OD260/280 ratio of 2.15 and an OD260/230 ratio of 2.39. The quality of the total RNA samples is shown in Figure 1a, which obviously shows that the extracted total RNA band type distribution was correct. The RNA quality met the requirements for database construction. The mRNA was purified by a magnetic bead method, and the length of the obtained mRNA ranged from 250 to 2000 bp (Figure 1b), indicating that qualified mRNAs were obtained. The double-stranded cDNA was amplified by LD-PCR. The results showed that the molecular weight distribution of the purified cDNA was normal and that the homogenized concentration and purity data were consistent (Figure S1a,b); therefore, it could be used for the construction of a cDNA library.

### 2.2. Library Identification and Titer Determination

**Figure 2:**
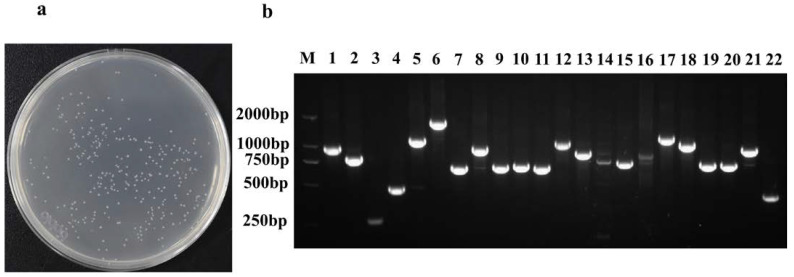
Yeast library identification and titer determination. (**a**) Diagram of the library titer test. (**b**) Library identification of PCR products via agarose gel electrophoresis; markers (2000/1000/750/500/250 bp); Lanes 1–22: PCR products.

The SmaI-linearized pGADT7-Recexpression vector and the purified double-stranded cDNA were co-transformed into DH10B hosts to obtain DH10B library bacteria (Figure 2a). The titer of the sandalwood cDNA library was 1.75 × 10^7^ CFU/mL, which guaranteed the reliability of the cDNA library screening results. Agarose gel electrophoresis of the monoclonal colony PCR products showed that 80% of the inserted fragments identified by PCR were over 750 bp in length, and the positivity rate of the library was greater than 90% (Figure 2b).

### 2.3. Promoter Prediction Results

We used the PlantCARE database to analyze the cis-acting elements of the *SaSSY* gene promoter (Table 1), and the results showed that a total of 31 cis-acting regulatory elements were predicted. These included the following: regulatory elements composed of a CAAT-box and a TATA-box, light-responsive element ATCT-motif, Box 4, G-Box, GT1-motif, I-box, and TCT-motif; a TGACG-motif and a CGTCA-motif, cis-acting regulatory elements involved in MeJA responsiveness; ABRE, a cis-acting element involved in the abscisic acid responsiveness; LTR, a low-temperature response control element; gibberellin response elements P-box and TATC-box; circadian, the regulatory element involved in the control of the biological clock; and regulatory element GCN4-motif, which is involved in endosperm expression. The remaining cis-acting regulatory elements were unnamed and functionally unknown control elements. We hypothesized that the promoter region of the *SaSSY* gene has the structural basis for potential regulatory binding.

### 2.4. Construction of Bait-Reporter Strains and Determination of the AbA Concentration

**Figure 3:**
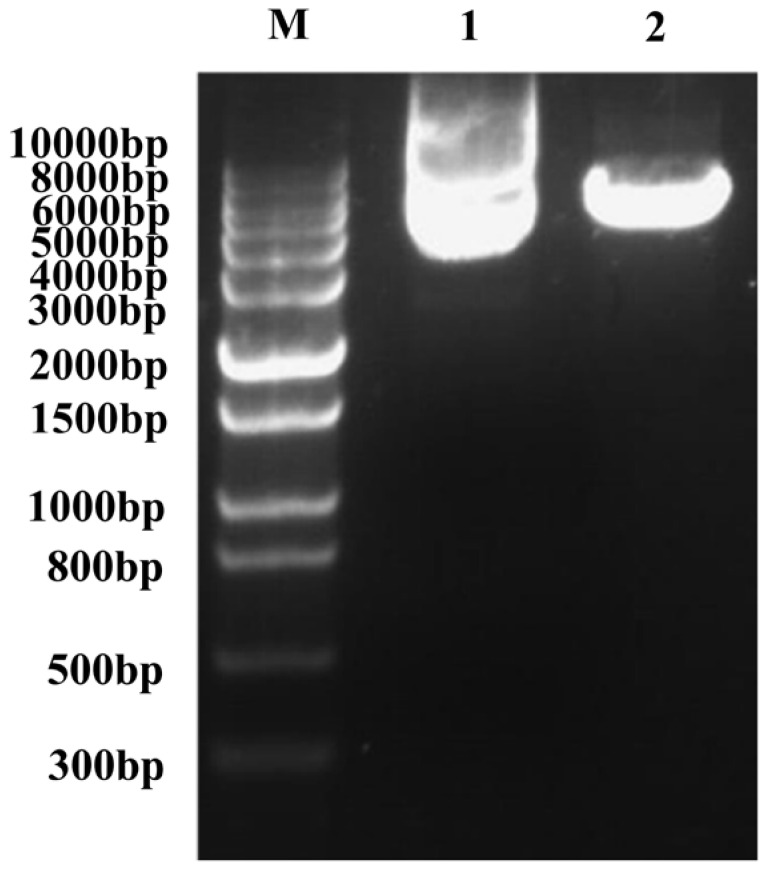
Agarose gel electrophoresis of the linearized bait carrier product. Marker (10,000/8000/6000/5000/4000/3000/2000/1500/1000/800/500/300 bp); Lane 1: pAbAi-Sal3G10690 plasmid; Lane 2: linearized pAbAi-Sal3G10690 plasmid.

**Figure 4:**
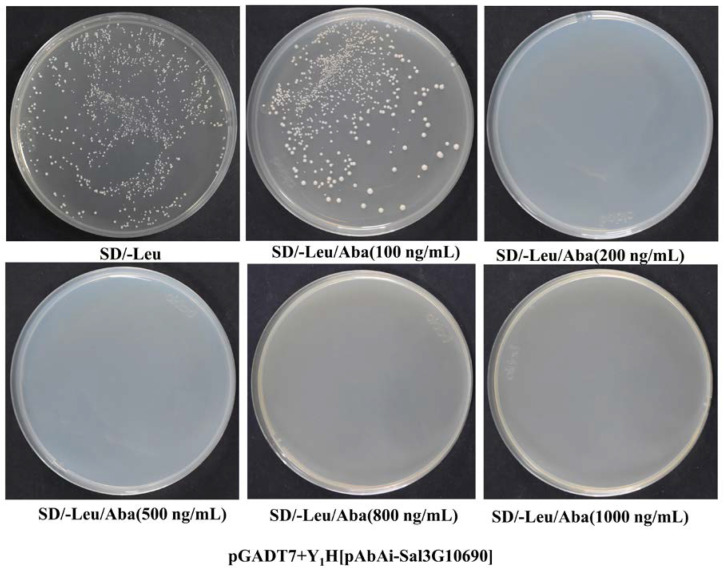
Determination of the minimum Aba concentration of positive clones of the bait vector.

The obtained recombinant plasmid pAbAi-Sal3G10690 was linearized and sampled for agarose gel electrophoresis assessment (Figure 3). The recombinant bait vectors (pAbAi-Sal3G10690) were transformed into Y1H Gold. Clear colonies can be seen on the SD/-Leu plate, indicating that the recombinant bait vector has been transformed into yeast cells. To avoid endogenous yeast transcription factors for target sequence recognition, we measured the minimum concentration of AbA that inhibits self-activation. In Figure 4, the minimum concentration of AbA required to inhibit the basic expression of *Sal3G10690* was 200 ng/mL.

### 2.5. Transformation of the Plasmid Library and Screening Results of Positive Clones

**Figure 5:**
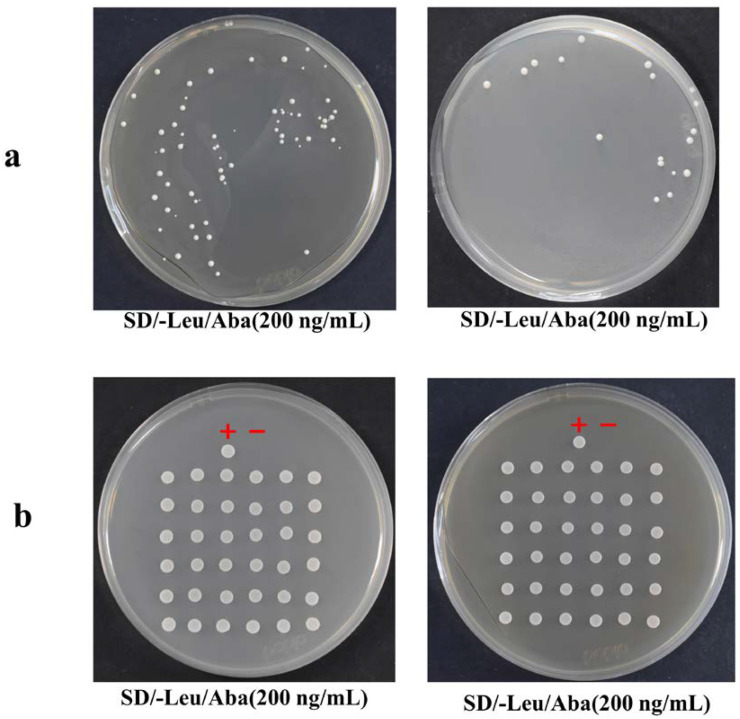
(**a**) Co-transformation screening of yeast cells coated on a SD/-Leu/Aba (200 ng/mL) plate. (**b**) Diagram of a positive clone screening point SD/-Leu/Aba (200 ng/mL) plate. Positive control +: pGADT7-rec-53+Y1H[pAbAi-p53]; Negative control −: pGADT7+Y1H[pAbAi-p53].

**Figure 6:**
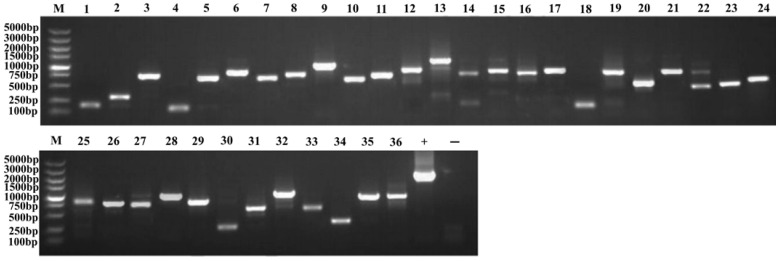
Agarose gel electrophoresis of PCR products from positive clones of the first round of screening. Marker (5000/3000/2000/1500/1000/750/500/250/100 bp); Lanes 1–36: Colony PCR products; Lane +: Positive control (pGADT7-T template PCR); Lane −: Negative control (water template PCR).

**Figure 7:**
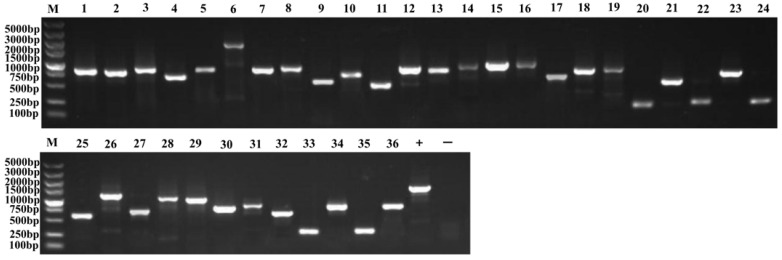
Agarose gel electrophoresis of PCR products from the second round of screening of the positive clones. Marker (5000/3000/2000/1500/1000/750/500/250/100 bp); Lanes 1–36: Colony PCR products; Lane +: Positive control (pGADT7-T template PCR); Lane −: Negative control (water template PCR).

The library plasmid was transformed into receptive cells of the bait strain (Figure 5a), and many positive clones were obtained by AbA screening (Figure 5b). PCR and agarose gel electrophoresis showed that the size of the inserted fragments varied, and the Prey plasmids contained in the positive clones had gene sizes ranging from 500 to 2000 bp (Figure 6 and Figure 7). After sequencing analysis and screening, 51 positive clones were successfully obtained (Table S2). Of these, through TAIR, we compared several transcription factors, including MYB, HSP20, MADS-box, and HD-ZIP. We used the NCBI database to predict the functions of the screened proteins (Table 2) and conducted a BLASTx/BLASTn comparison search on the resulting sequences. The results showed that we identified the transcription factor MYB36-like (Sal6G03620), small heat shock protein (Sal8G07920), hypothetical protein (Sal1G00910), and homeobox-leucine zipper protein ATHB-15 (Sal4G10880).

## 3. Discussion

The detection of sequence-specific regulatory transcription factor (TF) protein interaction with its DNA target site is generally performed by yeast one-hybrid screening [16]. In this study, a sandalwood *SaSSY* gene promoter library was successfully constructed. In addition, potential TF genes, such as *Sal6G03620* and *Sal8G07920*, were screened.

The gene expression regulatory network is composed of cis-reaction factors. There are 31 cis-regulatory elements upstream of the sandalwood *SaSSY* gene promoter. Among them, the P-box and TATC-box are gibberellin response elements, and gibberellin (GA) not only affects plant growth and development, but also participates in signal transduction. In higher plants, the upstream gibberellin signaling components and cis-acting factors that regulate downstream gibberellin response genes have been widely studied [17,18]. Studies have shown that foliar application of GA3 increases not only the expression level of monoterpene synthase genes but also the yield of monoterpenes. GA is responsible for the transcriptional regulation of monoterpene synthase genes [19]. In addition, gibberellins (GAs) are diterpenoid plant hormones essential for plant growth and development [20]. These findings can provide a reference for exploring whether sandalwood transcription factors can regulate enzymes related to terpenoid formation under the influence of the GA pathway via GA-responsive elements. The CGTCA motif and TGACG motif are MeJA response elements. Jasmonate, as a plant hormone, can induce the accumulation of many secondary metabolites by regulating jasmonic acid response transcription factors, such as in *Fagopyrum tataricum*, in which *FtJAZ1*, a key inhibitor of the jasmonic acid signaling pathway, specifically interacts with *FtMYB13* [21]. Previous studies have shown that the bHLH transcription factor MYC2 can positively regulate the terpenoid synthase *ASS1* under the influence of the jasmonic acid (JA) pathway [22]. The component analysis of promoters provides a reference for the identification of transcription factors that directly regulate *SaSSY* promoters by yeast one-hybrid. At the same time, it also lays a foundation for exploring the molecular mechanism of targeting transcription factors to regulate *SaSSY* expression.

Sal6G03620 was annotated to encode the transcription factor MYB36-like of sandalwood. MYB transcription factors represent families of proteins that contain conserved MYB DNA-binding domains. Compared with that of animals, the MYB protein subfamily of plants contains the R2R3-type MYB domain [23]. MYB transcription factors have been shown to play a key role in the biosynthesis of secondary metabolites in plants. In addition, MYB proteins also play a variety of roles in response to abiotic stresses such as drought, salt, and cold stress [24]. Studies have shown that the *MbMYB4* gene can enhance the cold and drought resistance of transgenic *Arabidopsis thaliana* plants [25]. In the poplar R2R3-MYB genome, most genes have drought-responsive expression patterns in different tissues [26]. In the sandalwood R2R3-MYB genome, 31 *R2R3-MYB* genes responded to cold treatment [27]. Previous studies have confirmed that *SlMYB11* can directly bind to the MYB site (CAACCA/TAACCA) of GPP, GLDH, and DHAR promoters in tomatoes [28]. Cis-acting element prediction analysis showed that the *SaSSY* promoter contained MYB transcription factor binding sites located at −1433, −1437, and −1791 (CAACCA/TAACCA) (Table 1). MYB transcription factors may serve as upstream regulatory factors for *SaSSY*, regulating the expression of the *SaSSY* gene.

Sal8G07920 was annotated as a small heat shock protein (HSP20) in sandalwood. HSPs are divided into five protein families based on molecular weight and sequence homology: HSP100s, HSP90s, HSP70s, HSP60s, and HSP20s. HSP20 is known as small HSP (sHSP) and has a molecular weight of approximately 12 to 42 kDa [29]. Heat shock protein 20 (HSP20) plays a vital role in plant growth and stress resistance, especially in enhancing plant stress resistance [30,31]. Relevant studies have shown that *AtHSP20* promotes the expression of AT3G30460 and helps Arabidopsis adapt to high-calcium environments by regulating ubiquitin-mediated protein degradation [32]. In the peach HSP20 genome, *PpHSP20-32* promotes plant growth and improves heat resistance. The plant height of Arabidopsis thaliana strains overexpressing *PpHSP20-32* was significantly greater than that of the WT. The seeds of the *PpHSP20-32*-overexpressing strain treated with high temperatures exhibited greater heat resistance [33]. Previous studies have shown that *Hsp20* is the target of *DnrH* in heat stress response, and *DnrH* enhances heat resistance by increasing the transcription of *Hsp20* mRNA [34]. However, whether HSP transcription factors regulate *SaSSY* gene expression still needs to be further researched.

Sal1G00910 was annotated as a hypothetical protein of sandalwood. The MADS-box gene family can be divided into two lineages, type I and type II, according to their protein domains. Type I genes are a heterogeneous population encoding only the 180 bp common DNA sequence of the MADS domain, while type II genes include well-studied homologous flower genes as well as other genes involved in various developmental processes, such as embryogenesis, flowering timing, and fruit development [35]. Related studies have identified a MADS-box family gene, *PtrANR1*, encoding an anthocyanin reductase in triloba orange and have shown that it can enhance plant drought resistance by promoting root development, increasing proline accumulation, and removing reactive oxygen species [36]. In papaya, *MADS* (*CpAGL18*) can bind the CarG-box (CC(A/T)6GG) element in the promoter of *CpACS1* and *CpSAUR32* [37]. However, the binding site of MADS-box transcription factor and *SaSSY* promoter still needs to be further explored.

Sal4G10880 was annotated as a homeobox-leucine zipper protein (ATHB-15) in sandalwood. The HD-ZIP gene family is not only a key player in crop improvement but is also involved in plant development and stress response [38]. In *Arabidopsis thaliana*, genetic systems composed of the HD-Zip II and HD-Zip III genes cooperate to establish bilateral symmetry along the paraxial to distal axes in the embryo and to control stem tip meristem activity [39]. A study revealed that the *PsnHDZ63* gene in transgenic *Populus simonii × P. IGra* plays an important role in salt tolerance [40]. In *Lilium longiflorum*, the *HD-Zip I* gene (*LlHB16*) directly binds to the promoter of *LlHSFA2* and activates its expression, and its binding site is TTCCAATCAACAAT [41]. However, the same binding site was not found in the *SaSSY* promoter.

The GO annotation results show that these candidate genes may mainly participate in biological processes such as cellular processes, response to stimulus, and metabolism (Figure S2). The KEGG annotation results show that these candidate genes may play a regulatory role in pathways such as carbohydrate metabolism, amino acid metabolism, and terpenoids and polyketides metabolism (Figure S3). These bioinformatics analysis results provide a reference for further mining candidate genes. To explore the molecular regulatory mechanism of *SaSSY*, a key gene that influences the synthesis of sandalwood terpene compounds, additional regulatory factors directly or indirectly involved in the synthesis of terpene compounds need to be explored. Additionally, the biological functions of candidate regulatory genes remain to be validated. This research offers a novel perspective on the transcriptional regulation of the *SaSSY* gene and establishes a solid foundation for the further exploration of the regulatory mechanisms governing sandalwood terpene synthesis.

## 4. Materials and Methods

### 4.1. Experimental Material

The experimental materials were grown in the greenhouse of the Institute of Tropical Forestry, Chinese Academy of Forestry (ambient light; temperature, 25 °C; relative humidity, 75%). The root, stem, and leaf tissues of the sandalwood seedlings were collected as samples and frozen in liquid nitrogen at −80 °C.

### 4.2. RNA Extraction and mRNA Purification

The total RNA of the sandalwood was extracted by the TRIzol method, the total RNA was assessed by agarose gel electrophoresis, and the quality and concentration of the total RNA were determined by a NanoDrop 2000C (Thermo Nanodrop2000C, Wuhan, China) [42]. Finally, mRNA was purified by a magnetic bead method.

### 4.3. Purification and Homogenization of Double-Stranded cDNA

The first cDNA strand was synthesized by reverse transcription using mRNA as a template, and double-stranded cDNA was synthesized by long-distance PCR (LD-PCR). After the reaction, 7 μL of the reaction product was subjected to agarose gel electrophoresis to assess the cDNA synthesis, and the remaining portion was purified. The cDNA was purified with a CHROMA SPIN TE-400 chromatographic column and then homogenized with DSN enzyme [43]. One microliter of cDNA was obtained for agarose gel electrophoresis to assess the homogenization.

### 4.4. Construction of a cDNA Library

A sandalwood cDNA library was constructed according to the instructions of the cDNA library construction kit (Takara Biomedical Technology (Beijing) Co., Ltd., Clontech, Beijing, China). Then, 7 μL purified double-stranded cDNA and 3 μL linearized pGADT7-Recexpression vector were initiated for homologous recombination by the in-fusion enzyme. Then, it was transformed into the DH10B host to obtain DH10B library bacteria. A total of 10 μL of the *E. coli* cDNA library solution was diluted to 1/10,000, and 200 μL of the diluted solution was spread on LB solid plates containing ampicillin, which were cultured in an inverted position at 37 °C for 16 h [44]. The number of colonies was counted, and the cDNA library titer was calculated. The calculation formula was as follows: clone number = library titer (CFU · mL^−1^) × slab volume (mL) × dilution factor.

### 4.5. Promoter Prediction

The *SaSSY* gene information of sandalwood was obtained from the reference genome sequence of sandalwood determined by Guangzhou Institute of Tropical Forestry. PlantCARE (https://bioinformatics.psb.ugent.be/webtools/plantcare/html/, accessed on 25 March 2024) was used to predict the upstream 2000 bp promoter cis-acting element of *SaSSY* gene [45].

### 4.6. Preparation of Receptive Cells

Y1H one-yeast colonies were selected from YPDA plates, inoculated into YPDA liquid media, and oscillated until the OD600 reached 0.15–0.3. The mixture was transferred to YPDA liquid medium, shaken until the culture reached an OD600 of 0.4–0.5, and centrifuged at room temperature. The yeast cells were treated with deionized water and 1.5 mL of TE/LiAc to obtain receptive Y1H yeast cells.

### 4.7. Transformation of Linearized Bait Plasmids into Yeast Cells

The recombinant plasmid (pAbAi-Sal3G10690) was digested with Bstb I/BstB I. We used the PEG/LiAc method to integrate pAbAi-Sal3G10690 into Y1H Gold and cultured it in solid SD/-Leu medium. The empty pAbAi vector was used as a positive control. Then, the recombinant yeast was screened on SD/-Leu medium with different concentrations of Aba and incubated at 30 °C for 3 days to determine the lowest concentration of AbA that could completely inhibit the strain growth for subsequent library screening.

### 4.8. Screening of the Y1H Library and Extraction of the Prey Plasmids

A cDNA library (25 μg) was transformed into yeast. The yeast solution was then resuspended with 0.9% NaCl and 100 μL of the suspension was spread on SD/-Leu medium containing AbA for 3 days. The number of colonies was counted, and the number of screened clones was calculated as follows: number of screened clones = number of clones × dilution times × suspension volume/coating volume. All positive clones were selected and inoculated on SD/-Leu/Aba liquid media. After oscillating the culture overnight, the yeast plasmid was extracted with a small amount of yeast extraction kit (Wuhan GeneCreate Biological Engineering Co., Ltd., Wuhan, China). Then, PCR was performed with universal primers, and the amplified products were assessed by agarose gel electrophoresis. The amplified samples with positive bands were sequenced with universal primers (Table S1), and the sequencing results were analyzed by sequence alignment.

## 5. Conclusions

Through the construction of a sandalwood cDNA library and the yeast one-hybrid technique, four potential regulatory genes that bind to the *SaSSY* promoter region were identified and found to have known or predicted functions in plant growth, stress resistance, signal transduction, transcriptional regulation, protease activity, and other candidate regulatory genes. Although most of the predicted gene functions were not directly related to *SaSSY* gene regulation of terpene formation, the candidate genes identified in this study form the basis for further exploration of the molecular mechanism of *SaSSY* transcription and expression level control of terpene formation.

## Figures and Tables

**Table 1:** Cis-acting regulatory elements of the *SaSSY* gene promoter.

Site Name	Sequence	Position	Function	Number of Repeats
AAGAA-motif	gGTAAAGAAA	−302	Function unknown	1
ABRE	ACGTG	+1840	cis-acting element involved in the abscisic acid responsiveness	1
as-1	TGACG	+1336	Function unknown	1
AT~TATA-box	TATATA	+442, +444, +446, +448, +450, +452, +454, +456, +458, +888, −1259, −1537, −1906, −1908	Function unknown	14
ATCT-motif	AATCTAATCC	−26	cis-acting element involved in light responsiveness	1
Box 4	ATTAAT	+336, +354, +1042	cis-acting element involved in light responsiveness	3
CAAT-box	CAAAT	−22, +53, +221, −227, −317, +592, +680, +899, +907, +1353, −1356, +1700, +1780	common cis-acting element in promoter and enhancer regions	13
CAAT-box	CAAT	+48, −157, +164, +273, +323, −327, −463, −635, −657, −683, −722, +749, −952, +985, −1204, +1224, −1297, −1317, +1625, +1781, +1878	Function unknown	21
CGTCA-motif	CGTCA	−1336	cis-acting regulatory element involved in the MeJA responsiveness	1
circadian	CAAAGATATC	−404	cis-acting regulatory element involved in circadian control	1
ERE	ATTTTAAA	+1640	Function unknown	1
G-Box	CACGTT	−1839	cis-acting regulatory element involved in light responsiveness	1
GCN4_motif	TGAGTCA	+1421	cis-regulatory element involved in endosperm expression	1
GT1-motif	GGTTAA(T)	−548, −1113, −1114, +1792	Light-responsive element	2
I-box	AAGATAAGGCT	−555	part of a light-responsive element	1
LTR	CCGAAA	+1818	cis-acting element involved in low-temperature responsiveness	1
MYB	CAACCA	−1433, −1437	Function unknown	2
MYB-like	TAACCA	−1791	Function unknown	1
Myc	TCTCTTA	−1087	Function unknown	1
MYC	CAT(T/G) TG	+316, −810, −1700, +1477	Function unknown	2
P-box	CCTTTTG	+1931	gibberellin-responsive element	1
STRE	AGGGG	−1140, −1929	Function unknown	2
TATA	TATAAAAT	−575, +1763	Function unknown	2
TATA-box	AT(T)ATA(A/T)/TATA(AAAA)/TATATA(A)/TATTTAAA	−341, −578, −1675, −1980, +340, +440, +886, +342, +379, +428, +460, +579, +890, +911, −1082, −1095, −1261, −1491, −1539, −1676, −1763, −1910, −1981, +378, +443, +445, +447, +449, +451, +453, +455, +457, +459, −1094, −1258, −1536, −1907, −441, −887, +442, +444, +446, +448, +450, +452, +454, +456, +458, +888, −1259, −1537, −1906, −1908, −576, −1978, −577, −1979, +1081, +1399	core promoter element around −30 of transcription start	59
TATC-box	TATCCCA	+1727	cis-acting element involved in gibberellin responsiveness	1
TCT-motif	TCTTAC	+123, −706	part of a light-responsive element	2
TGACG-motif	TGACG	+1336	cis-acting regulatory element involved in the MeJA responsiveness	1
Unnamed-1	CGTGG	+1841	Function unknown	1
Unnamed-16	GCTGCCCGTC	−1864	Function unknown	1
Unnamed-4	CTCC	+83, +201, −743, −781, +948, −962, +1153, −1360, +1735	Function unknown	9
WRE3	CCACCT	+1660, −1871	Function unknown	2

**Table 2:** Screening results of the *SaSSY* promoter transcription factors.

Name	Fragment Length (bp)	Blastx/Blastn	NCBI Number	Description	Scientific Name
Sal6G03620.1	1153/744	Blastn	XM_058111947.1	PREDICTED: Malania oleifera transcription factor MYB36-like (LOC131157642), mRNA	*Malania oleifera*
Sal8G07920.2	1225/666	Blastn	XM_058095332.1	PREDICTED: Malania oleifera small heat shock protein, chloroplastic (LOC131146053), mRNA	*Malania oleifera*
Sal1G00910.1	597/206	Blastx	KAI6692205.1	hypothetical protein NL676_019915	*Syzygium grande*
Sal4G10880.1	2514/836	Blastx	XP_057976689.1	homeobox-leucine zipper protein ATHB-15	*Malania oleifera*

## Data Availability

Data are contained within the article and Supplementary Materials.

## Supplementary Materials

The following supporting information can be downloaded at: https://www.mdpi.com/article/10.3390/plants13131882/s1, Figure S1: Agarose gel electrophoresis of double-strand cDNA; Figure S2: the GO Pathway enrichment analysis; Figure S3: the KEGG Pathway enrichment analysis; Table S1: The primer sets used in this study; Table S2: Information on genes that interacted with *SaSSY* by yeast one-hybrid screening.
