# Supplementary material for: Identification of Transcription Factors of Santalene Synthase Gene Promoters and SaSSY Cis-Elements through Yeast One-Hybrid Screening in Santalum album L
Source: Plants (Basel). 2024 Jul 8;13(13):1882. doi: 10.3390/plants13131882 (PMC11244121; doi:10.3390/plants13131882)
Supplement: Supplementary file 1 [file plants-13-01882-s001.zip › Figure S1.pdf]

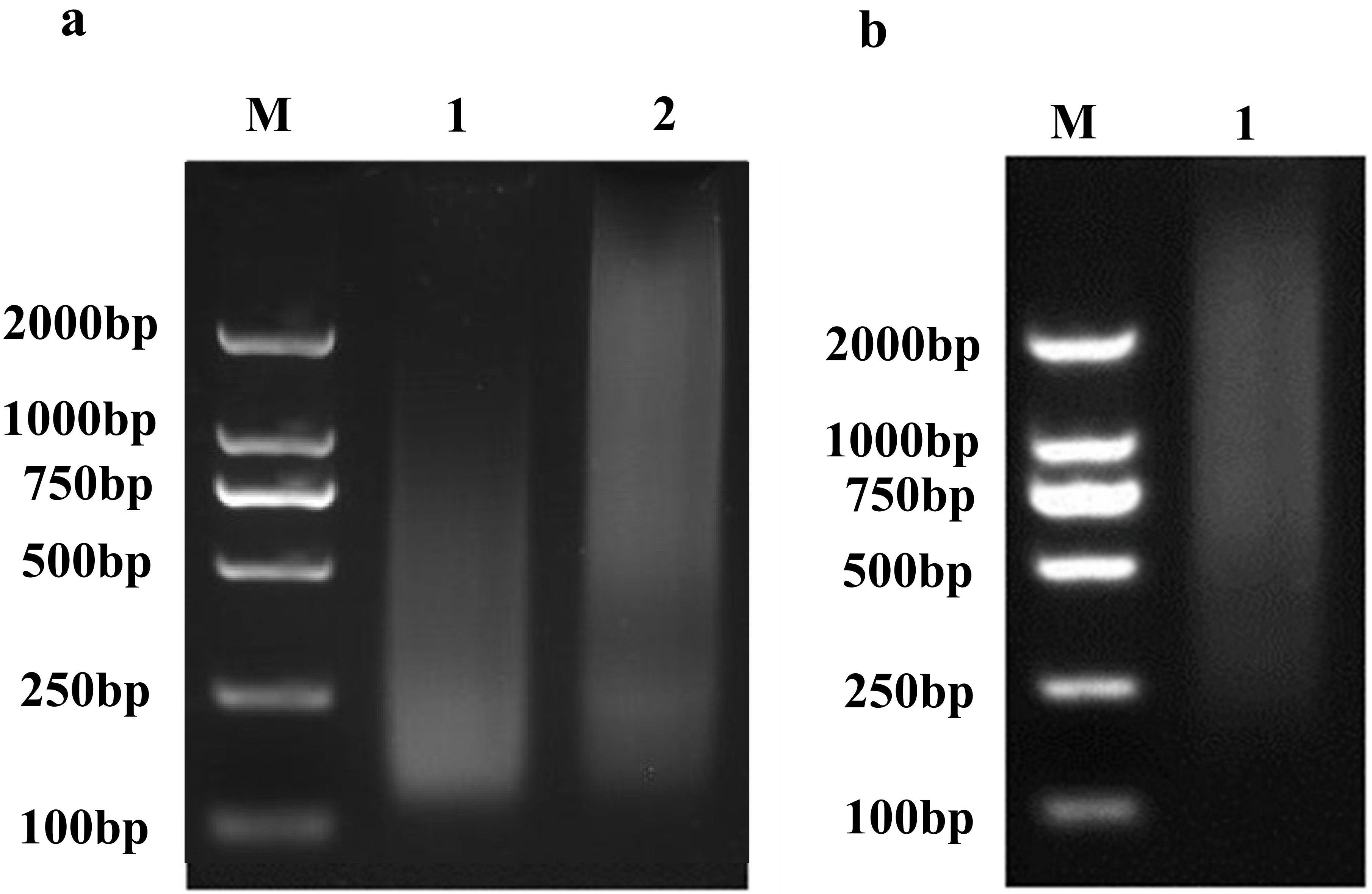

Figure S1. Agarose gel electrophoresis of double-strand cDNA. (a) Agarose gel electrophoresis of double-stranded cDNA by LD-PCR; marker (2000/1000/750/500/250/100 bp); Lanes 1-2: purification of ds cDNA. (b) Agarose gel electrophoresis of homogenized double-stranded cDNA; marker (2000/1000/750/500/250/100 bp); Lane 1: homogenized ds cDNA.
