# Supplementary figures and images for: Identification of Transcription Factors of Santalene Synthase Gene Promoters and SaSSY Cis-Elements through Yeast One-Hybrid Screening in Santalum album L
Source: Plants (Basel). 2024 Jul 8;13(13):1882. doi: 10.3390/plants13131882 (PMC11244121; doi:10.3390/plants13131882)

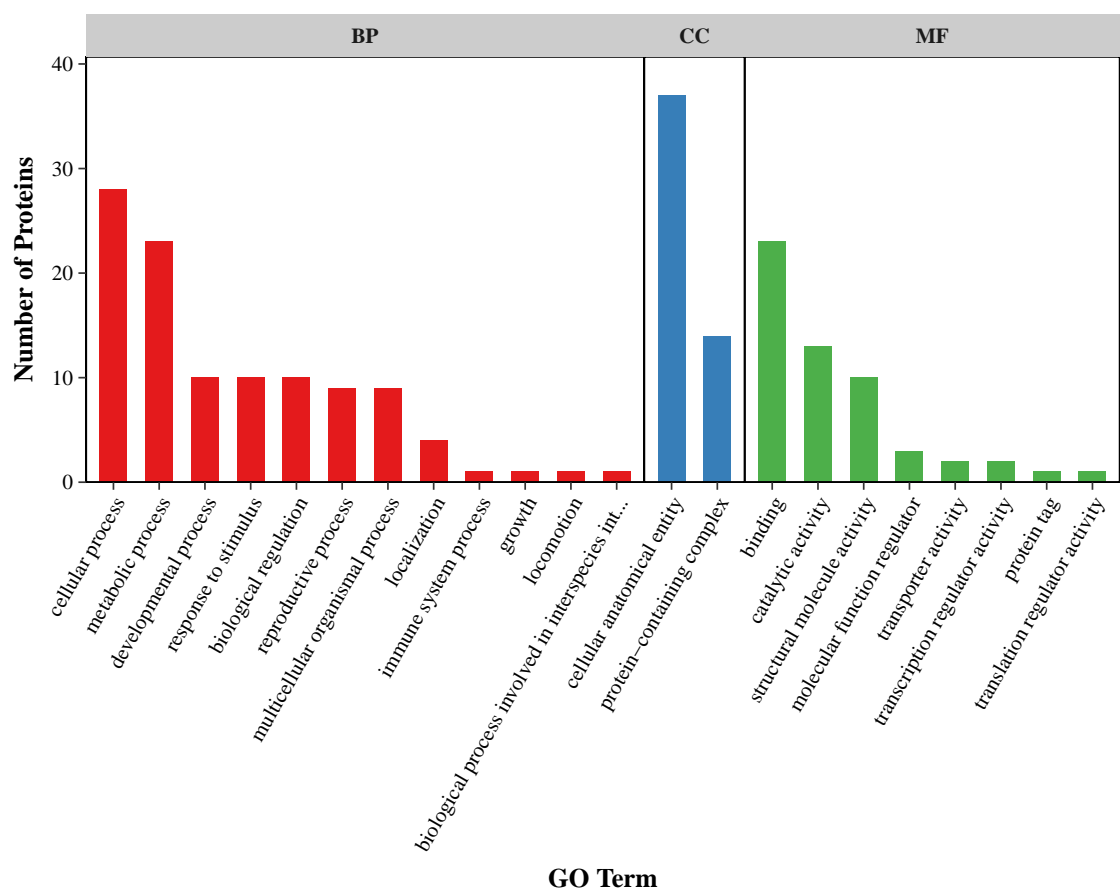

Supplement: Supplementary file 1 [file plants-13-01882-s001.zip › Figure S2.pdf]

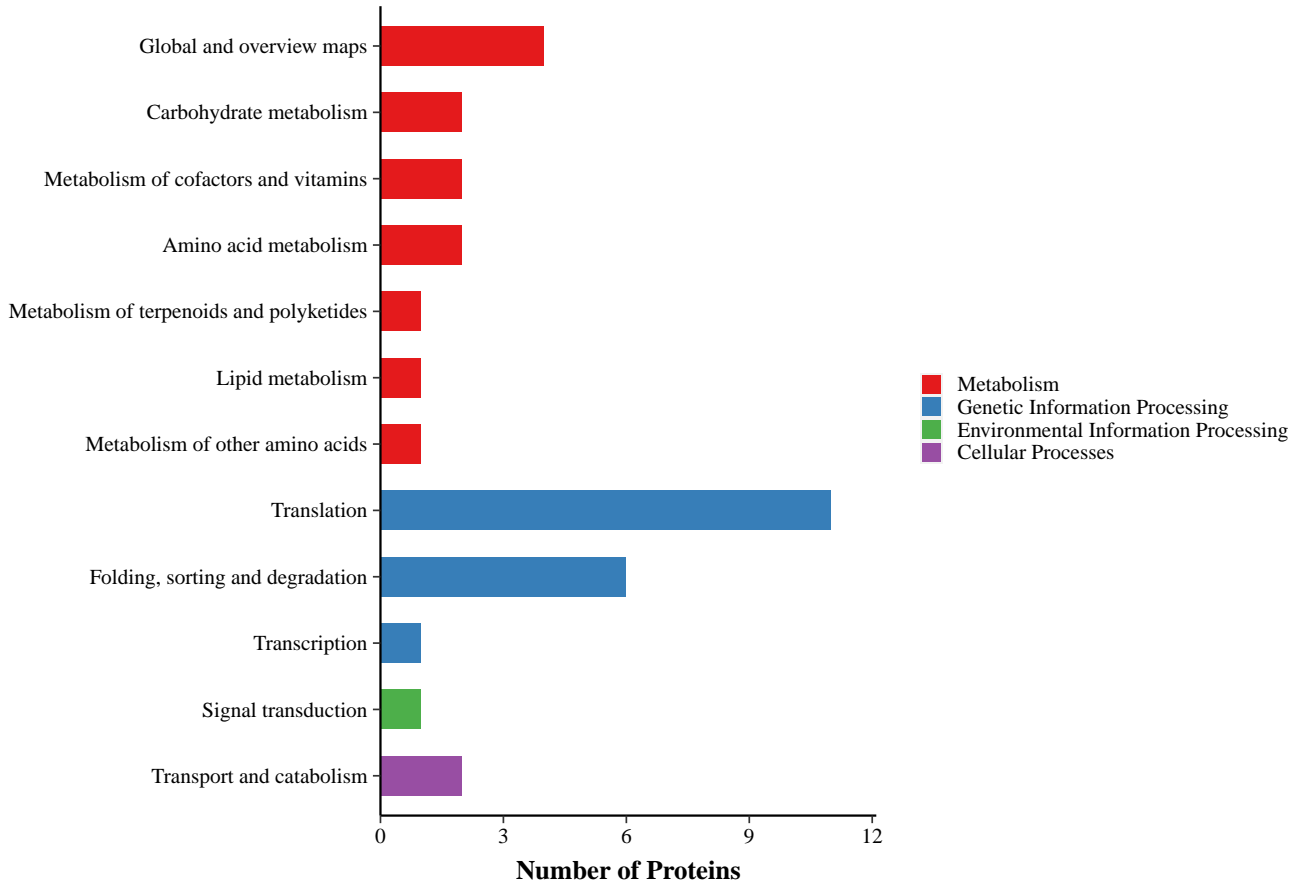

Supplement: Supplementary file 1 [file plants-13-01882-s001.zip › Figure S3.pdf]
